# The association between weight-adjusted waist index and respiratory symptoms in U.S adults: A national cross-sectional study

**DOI:** 10.1371/journal.pone.0322013

**Published:** 2025-04-30

**Authors:** Yu Wang, Wenlu Chang, Yiwei Lu, Yi Xin, Ximing Li

**Affiliations:** 1 Department of Nutrition, Chest Hospital, Tianjin University, Tianjin, China; 2 Tianjin Key Laboratory of Cardiovascular Emergency and Critical Care, Tianjin Municipal Science and Technology Bureau, Tianjin, China; Jordan University of Science and Technology Faculty of Computer and Information Technology, JORDAN

## Abstract

**Objective:**

This study aimed to evaluate the relationship between the weight-adjusted waist index (WWI) and respiratory symptoms, including cough, wheezing, and dyspnea, as well as the related respiratory diseases, namely chronic obstructive pulmonary disease (COPD) and asthma, in adults.

**Methods:**

This cross-sectional study included 14,760 adults aged over 40 years, drawn from the National Health and Nutrition Examination Survey (NHANES) conducted between 2003 and 2012. Weighted logistic regression analysis was employed to investigate the association between WWI and respiratory symptoms, including cough, wheezing, and dyspnea, as well as related respiratory diseases such as COPD and asthma. Subgroup analyses and interaction tests were performed to stratify the data by age, gender, and race. Additionally, smooth curve fitting and threshold effect analyses were utilized to explore potential non-linear relationships between WWI and respiratory symptoms, as well as the associated respiratory diseases.

**Results:**

After adjusting for covariates, a positive association was observed between WWI and respiratory symptoms, including cough, wheezing, and dyspnea [odds ratio (OR): 1.39, 95% confidence interval (CI): 1.29–1.50; OR: 1.62, 95% CI: 1.51–1.73; OR: 1.58, 95% CI: 1.50–1.67]. This association extended to related respiratory diseases such as COPD and asthma (OR: 1.42, 95% CI: 1.30–1.54; OR: 1.43, 95% CI: 1.33–1.54). Subgroup analyses indicated that the relationship between WWI and wheezing was modified by race, whereas dyspnea was influenced by age, gender, and race. For COPD, the association was affected by gender. Smoothed curve fitting revealed nonlinear, J-shaped associations between WWI and cough, COPD, and asthma (OR: 1.39, 95% CI: 1.29–1.50, P < 0.001; OR: 1.42, 95% CI: 1.30–1.54, P < 0.001; OR: 1.43, 95% CI: 1.33–1.54, P < 0.001). Furthermore, the breakpoint (K) was found to be 9.99 for both wheezing and dyspnea, with P < 0.05 for log-likelihood ratios in both instances.

**Conclusions:**

This study provides evidence linking elevated levels of WWI to an increased risk of respiratory symptoms, including cough, wheezing, and dyspnea, as well as associated respiratory diseases such as COPD and asthma in U.S adults. These findings offer novel insights into the management of respiratory symptoms and diseases.

## Introduction

Patients can easily recognize common respiratory symptoms, including cough, wheezing, and dyspnea, which are hallmark indicators of conditions such as chronic obstructive pulmonary disease (COPD) and asthma [1,2]. These symptoms not only signify the presence of underlying chronic conditions but also represent a substantial threat to both physical and mental health [3,4]. Worldwide, respiratory diseases such as pneumonia, lung cancer, COPD, and asthma rank among the leading causes of disability and mortality [5–7]. More than half of the global adult population reports experiencing at least one respiratory symptom associated with these diseases [8–10], significantly affecting quality of life [11].

According to the World Health Organization, over one-third of American adults are classified as obese [12]. Research indicates that obesity is a significant risk factor and predictor of adverse health outcomes [13]. It is associated with a shortened life expectancy and a markedly increased risk of diseases, including dyslipidemia, hypertension, and hyperglycemia [14,15], particularly cardiovascular and pulmonary disorders [16,17]. The impact of obesity on respiratory health is influenced by various factors, including structural changes (such as a reduction in total lung capacity) and biochemical alterations (notably the deregulation of cytokines and adipokines) [18,19]. The mechanical effects of obesity restrict airway function, increase breathing resistance, and frequently result in dyspnea and wheezing. Moreover, obesity disrupts adipokine secretion and homeostasis, thereby facilitating the onset and progression of lung disease [19]. As a consequence, many obese patients experience respiratory symptoms and associated respiratory diseases.

In a recent study investigating the relationship between obesity and respiratory symptoms, researchers employed body mass index (BMI) to evaluate participants’ obesity status. The study conducted by Sun et al. in the United States among individuals aged over 40 revealed a U-shaped association between BMI and respiratory symptoms, including cough, wheezing, dyspnea, asthma, and COPD [20]. However, it is essential to acknowledge the phenomenon known as the “obesity paradox” [21]. This unexpected paradox may be attributed to BMI’s inability to distinguish between muscle and fat mass. Currently, BMI is primarily used to assess central obesity and is regarded as a crude indicator of obesity predisposition [22]. The weight-adjusted waist index (WWI), proposed in 2018 [23], offers a more precise measure of fat and muscle mass composition. It primarily indicates central obesity and operates independently of body weight [24]. In a multi-ethnic study on atherosclerosis, WWI demonstrated positive correlations with abdominal fat measures and negative correlations with abdominal muscle mass measures, regardless of race or ethnicity [25]. This metric has shown a distinct advantage over BMI in the development of diseases across various systems [26]. These findings further emphasize the reliability and potential of WWI [25]. Nevertheless, no studies have investigated the association between WWI and respiratory symptoms, as well as the related respiratory diseases.

Consequently, our study aimed to investigate the association between WWI and respiratory symptoms—including cough, wheezing, and dyspnea—as well as related diseases such as COPD and asthma, utilizing a nationally representative sample from the National Health and Nutrition Examination Survey (NHANES).

## Methods

### Study population

We conducted a cross-sectional study utilizing data from the NHANES spanning 2003–2012. NHANES, a national survey administered by the National Center for Health Statistics (NCHS), aims to evaluate the nutrition and health status of the U.S population. The procedures employed in NHANES were approved by the NCHS Research Ethics Review Board (ERB). The portions of this study involving human participants, human materials, or human data were conducted in accordance with the Declaration of Helsinki. The participants provided their written informed consent to participate in this study. Comprehensive methodologies and results from NHANES can be accessed at www.cdc.gov/nchs/nhanes/.

**Fig 1:**
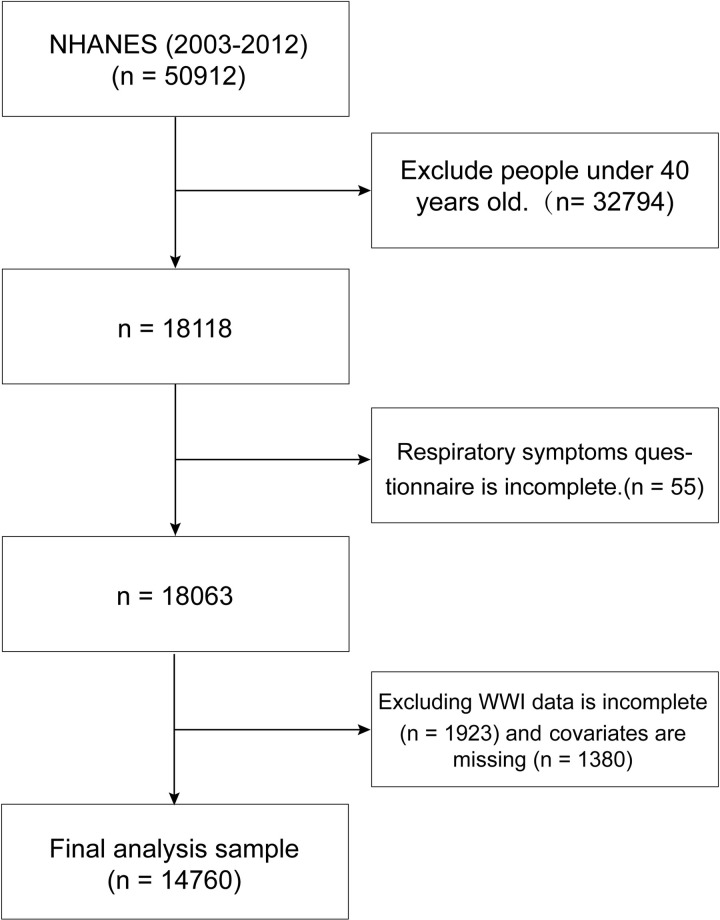
Flow chart of the study population (NHANES 2003–2012).

The study population consisted of 18,118 participants aged over 40 years, derived from NHANES 2003–2012. We excluded participants with missing data from the respiratory symptom questionnaire (n = 55) and those lacking WW data (n = 1,923). Furthermore, participants with missing covariate data (n = 1,380) were also excluded. Consequently, the final sample comprised 14,760 participants (see Fig 1).

### Evaluation of weight-adjusted waist index

The WWI, a novel metric designed to predict central obesity, is determined by dividing the waist circumference (WC, in cm) by the square root of body weight (in kg). A higher WWI score indicates a greater degree of obesity. Trained health technicians collected body measurements, including weight and WC, at the mobile examination center (MEC). The WWI for each participant was calculated by dividing the WC in centimeters by the square root of the weight in kilograms, with the result rounded to two decimal places [27]. WWI was treated as a continuous variable, and individuals were subsequently stratified into quartiles for analysis. In our study, WWI served as the exposure variable.

### Evaluation of respiratory symptoms and the associated respiratory diseases

Cough was confirmed based on a positive response to the question: “Do you usually cough on most days for 3 consecutive months or longer each year?” Wheezing and dyspnea were confirmed through affirmative responses to the questions: “In the past 12 months, have you experienced wheezing or whistling in your chest?” and “Have you had shortness of breath when hurrying on level ground or when walking up a slight hill?” The diagnosis of COPD was based on a positive response to the question: “Has a doctor or other health professional diagnosed you with chronic bronchitis or emphysema?” Similarly, the diagnosis of asthma was determined by a positive response to the question: “Has a doctor or other health professional diagnosed you with asthma?”[20].

### Selection of covariates

We incorporated demographic covariates, including age (in years), gender (male/female), race (Mexican-American, other Hispanic, non-Hispanic Black, non-Hispanic White), educational level (less than high school, high school, greater than high school), marital status (married/living with a partner, divorced/separated/widowed, never married), the ratio of income to poverty (PIR), and smoking status (having smoked more than 100 lifetime cigarettes) [20].

### Statistical analyses

According to the guidelines of the Centers for Disease Control and Prevention (CDC), all statistical analyses employed NHANES sampling weights, which accounted for the complex sampling design involving multiple stages of cluster surveys [28]. Continuous variables were summarized using either the mean ± standard deviation or the median (interquartile range), depending on their distribution, while categorical variables were expressed as counts (percentages). We utilized chi-square and t-tests to assess differences between participants grouped by WWI quartiles. Weighted logistic regression analyses were conducted to investigate the association between WWI and respiratory symptoms, COPD, and asthma. Three models were developed to ensure the stability of the results after adjusting for various covariates. Model 1 included no covariate adjustments. Model 2 adjusted for age, gender, and race. Model 3 adjusted for age, gender, race, education level, marital status, PIR, and smoking status. Subgroup analyses were performed to explore the association between WWI and respiratory symptoms, COPD, and asthma across specific populations, considering variables such as age, gender, and race. Interaction tests were conducted to assess the consistency of correlations among subgroups, with interaction P-values calculated using interaction terms included in the logistic regression models. Smooth curve fitting and threshold effect analyses were employed to investigate the potential non-linear relationship between WWI and respiratory symptoms (cough, wheezing, and dyspnea), as well as the associated respiratory diseases (COPD and asthma). Analyses were performed using R (version 4.2) or Empowerstats (version 2.0), with statistical significance defined as P < 0.05 (two-sided).

## Results

### Baseline characteristics

The study involved 14,760 participants aged over 40 years, with a mean age of 59.70 years and a standard deviation of 12.66 years. Among these participants, 49.8% were male and 50.2% were female. The mean WWI for all participants was 11.24, with a standard deviation of 0.77. The prevalence rates for cough, wheezing, dyspnea, COPD, and asthma were 10.9%, 14.5%, 35.0%, 8.8%, and 12.2%, respectively. Significant differences were found across age, gender, race, education level, marital status, PIR, and smoking status among the WWI quartiles (all P < 0.05) (see Table 1). Participants in the lowest WWI quartile (Q1) demonstrated higher education levels, a greater PIR, and more favorable marital statuses compared to those in the highest WWI quartile (Q4). Furthermore, the prevalence of asthma, COPD, dyspnea, cough, and wheezing increased with ascending WWI quartiles (see Table 1).

**Table 1:** Baseline characteristics of study population according to weight-adjusted waist index quartile.

Characteristics	Total, n	Weight-adjusted waist index				*P*-value
	14,760	Q 1	Q 2	Q 3	Q 4	
		(7.90–10.71)	(10.71–11.23)	(11.23–11.75)	(11.75–15.70)	
		N = 3,690	N = 3,690	N = 3,690	N = 3,690	
Age (years)		53.56 ± 11.03	57.65 ± 12.02	61.79 ± 12.14	65.80 ± 12.01	< 0.001
Gender, (%)						< 0.001
Male	7,344	54.7	55.2	51.5	37.7	
Female	7,416	45.3	44.8	48.5	62.4	
Race, (%)						< 0.001
Mexican American	2,251	8.5	15.0	18.0	19.5	
Other Hispanic	1,942	12.1	13.1	14.2	13.2	
Non-Hispanic White	7,440	49.3	50.4	49.4	52.6	
Non-Hispanic Black	3,127	30.1	21.5	18.5	14.7	
Education level, (%)						< 0.001
<high school	4,372	19.9	25.7	31.7	41.3	
High school	3,491	21.8	23.4	25.7	23.8	
> high school	6,897	58.4	51.0	42.7	35.0	
Marital status, (%)						< 0.001
Married/living with a partner	9,213	64.8	66.3	65.3	53.3	
Divorced/separated/widowed	4,435	26.0	26.7	28.1	39.3	
Never married	1,112	9.2	7.0	6.6	7.4	
PIR		3.04 ± 1.68	2.79 ± 1.62	2.57 ± 1.58	2.19 ± 1.47	< 0.001
Smoking, (%)						< 0.05
Yes	7,248	49.1	50.8	52.2	51.5	
No	7,512	51.0	49.2	47.8	48.5	
Comorbidities, (%)						
COPD	1,302	5.8	7.2	9.0	13.3	<0.001
Asthma	1,796	10.4	11.0	11.9	15.5	<0.001
Respiratory symptoms, (%)						
Cough	1,601	8.4	9.3	10.7	15.0	<0.001
Wheezing	2,145	11.2	12.3	15.3	19.3	<0.001
Dyspnea	5,166	24.3	31.0	37.1	47.6	<0.001

Mean ± SD for continuous variables: the P value was calculated by the weighted linear regression model. (%) for categorical variables: the P value was calculated by the weighted chi-square test. Abbreviation: PIR, ratio of income to poverty; COPD, chronic obstructive pulmonary disease; Q, quartile.

### Associations between WWI and respiratory outcomes

Multifactorial logistic regression analysis revealed a positive association between WWI, treated as a continuous variable, and the incidence of cough, wheezing, and dyspnea in the unadjusted Model 1. These associations remained statistically significant in Model 3 after adjusting for potential confounders, which included age, gender, race, education level, marital status, PIR, and smoking [odds ratio (OR): 1.39, 95% confidence interval (CI): 1.29–1.50, *P* < 0.001; OR: 1.62, 95% CI: 1.51–1.73, *P* < 0.001; OR: 1.58, 95% CI: 1.50–1.67, *P* < 0.001] (Table 2). When WWI was categorized into quartiles, participants in the highest quartile (Q4) exhibited significantly higher odds of respiratory symptoms compared to those in the lowest quartile (Q1), including cough (OR: 1.80, 95% CI: 1.53–2.12, *P* < 0.001), wheezing (OR: 2.35, 95% CI: 2.03–2.72, *P* < 0.001), dyspnea (OR: 2.42, 95% CI: 2.16–2.70, *P* < 0.001), COPD (OR: 2.00, 95% CI: 1.66–2.42, *P* < 0.001), and asthma (OR: 1.91, 95% CI: 1.63–2.23, *P* < 0.001) (Table 2).

**Table 2:** Association of weight-adjusted waist index and respiratory symptoms, COPD, and asthma.

	Model 1	*P*-value	Model 2	*P*-value	Model 3	*P*-value
OR (95% CI)	OR (95% CI)	OR (95% CI)
Cough						
WWI	1.42 (1.33, 1.52)	< 0.001	1.52 (1.41, 1.64)	< 0.001	1.39 (1.29, 1.50)	< 0.001
Q1	reference		reference		reference	
Q2	1.11 (0.95, 1.31)	0.1895	1.15 (0.98, 1.36)	0.0850	1.11 (0.94, 1.31)	0.2231
Q3	1.31 (1.12, 1.53)	< 0.001	1.42 (1.21, 1.67)	< 0.001	1.29 (1.10, 1.53)	< 0.01
Q4	1.93 (1.66, 2.23)	< 0.001	2.15 (1.83, 2.53)	< 0.001	1.80 (1.53, 2.12)	< 0.001
Wheezing						
WWI	1.42 (1.34, 1.51)	< 0.001	1.72 (1.61, 1.84)	< 0.001	1.62 (1.51, 1.73)	< 0.001
Q1	reference		reference		reference	
Q2	1.10 (0.96, 1.27)	0.1703	1.26 (1.09, 1.46)	< 0.01	1.23 (1.06, 1.42)	< 0.01
Q3	1.42 (1.24, 1.63)	< 0.001	1.82 (1.58, 2.10)	< 0.001	1.72 (1.49, 1.99)	< 0.001
Q4	1.88 (1.65, 2.14)	< 0.001	2.66 (2.31, 3.08)	< 0.001	2.35 (2.03, 2.72)	< 0.001
Dyspnea						
WWI	1.70 (1.63, 1.78)	< 0.001	1.68 (1.60, 1.77)	< 0.001	1.58 (1.50, 1.67)	< 0.001
Q1	reference		reference		reference	
Q2	1.40 (1.27, 1.56)	< 0.001	1.43 (1.29, 1.59)	< 0.001	1.40 (1.26, 1.55)	< 0.001
Q3	1.84 (1.66, 2.03)	< 0.001	1.86 (1.67, 2.06)	< 0.001	1.76 (1.58, 1.95)	< 0.001
Q4	2.84 (2.57, 3.13)	< 0.001	2.73 (2.45, 3.04)	< 0.001	2.42 (2.16, 2.70)	< 0.001
COPD						
WWI	1.58 (1.47, 1.70)	< 0.001	1.56 (1.44, 1.69)	< 0.001	1.42 (1.30, 1.54)	< 0.001
Q1	reference		reference		reference	
Q2	1.26 (1.04, 1.51)	< 0.05	1.28 (1.06, 1.54)	< 0.05	1.23 (1.02, 1.49)	< 0.05
Q3	1.61 (1.35, 1.93)	< 0.001	1.63 (1.35, 1.96)	< 0.001	1.51 (1.25, 1.82)	< 0.001
Q4	2.49 (2.10, 2.94)	< 0.001	2.40 (2.00, 2.87)	< 0.001	2.00 (1.66, 2.41)	< 0.001
Asthma						
WWI	1.29 (1.21, 1.38)	< 0.001	1.46 (1.36, 1.57)	< 0.001	1.43 (1.33, 1.54)	< 0.001
Q1	reference		reference		reference	
Q2	1.06 (0.92, 1.23)	0.4279	1.19 (1.03, 1.38)	< 0.05	1.18 (1.02, 1.38)	< 0.05
Q3	1.17 (1.01, 1.36)	< 0.05	1.42 (1.22, 1.66)	< 0.001	1.41 (1.21, 1.64)	< 0.001
Q4	1.59 (1.38, 1.82)	< 0.001	1.99 (1.71, 2.32)	< 0.001	1.91 (1.63, 2.23)	< 0.001

Model 1: no covariates were adjusted. Model 2: age, gender, and race were adjusted. Model 3: age, gender, race, education level, marital status, PIR, and smoking were adjusted. Abbreviation: WWI, weight-adjusted waist index; Q, quartile; COPD, chronic obstructive pulmonary disease; OR, odds ratio; CI, confidence interval.

### Subgroup analysis

Subgroup analyses and interaction tests were performed, stratified by age, gender, and race, to evaluate the consistency of the association between WWI and respiratory symptoms (Tables 3–5–5). Age significantly modified the association between WWI and dyspnea (P for interaction = 0.0383), with participants aged 60–69 years exhibiting a higher risk of cough and wheezing per unit increase in WWI (cough: OR = 1.61, 95% CI: 1.37–1.89, *P* < 0.001; wheezing: OR = 1.81, 95% CI: 1.57–2.08, *P* < 0.001) (Table 3). The association between WWI and dyspnea and COPD also demonstrated significant gender differences (Table 4), with males exhibiting a greater risk of cough and wheezing per unit increase in WWI (cough: OR = 1.43, 95% CI: 1.28–1.60, *P* < 0.001; wheezing: OR = 1.69, 95% CI: 1.53–1.87, *P* < 0.001). Race significantly influenced the association between WWI and wheezing and dyspnea (Table 5), with non-Hispanic Black participants demonstrating the highest risk of developing cough per unit increase in WWI (OR = 1.66, 95% CI: 1.40–1.98, *P* < 0.001).

**Table 3:** Association between weight-adjusted waist index and respiratory symptoms, COPD, and asthma by age.

	OR (95% CI)	*P-*value	*P* for interaction
Cough			0.2167
Age			
40–49	1.34 (1.15, 1.56)	< 0.001	
50–59	1.30 (1.12, 1.52)	< 0.001	
60–69	1.61 (1.37, 1.89)	< 0.001	
≥ 70	1.34 (1.16, 1.54)	< 0.001	
Wheezing			0.2477
Age			
40–49	1.56 (1.38, 1.77)	< 0.001	
50–59	1.57 (1.38, 1.80)	< 0.001	
60–69	1.81 (1.57, 2.08)	< 0.001	
≥ 70	1.50 (1.31, 1.72)	< 0.001	
Dyspnea			< 0.05
Age			
40–49	1.65 (1.49, 1.82)	< 0.001	
50–59	1.66 (1.49, 1.85)	< 0.001	
60–69	1.70 (1.53, 1.88)	< 0.001	
≥ 70	1.42 (1.29, 1.56)	< 0.001	
COPD			0.1351
Age			
40–49	1.43 (1.20, 1.71)	< 0.001	
50–59	1.41 (1.18, 1.69)	< 0.001	
60–69	1.60 (1.35, 1.89)	< 0.001	
≥ 70	1.23 (1.06, 1.42)	< 0.01	
Asthma			0.1863
Age			
40–49	1.46 (1.28, 1.67)	< 0.001	
50–59	1.26 (1.10, 1.45)	< 0.01	
60–69	1.56 (1.35, 1.80)	< 0.001	
≥ 70	1.37 (1.19, 1.59)	< 0.001	

Gender, race, education level, marital status, PIR, and smoking were adjusted. Abbreviation: COPD, chronic obstructive pulmonary disease; OR, odds ratio; CI, confidence interval.

**Table 4:** Association between weight-adjusted waist index and respiratory symptoms, COPD, and asthma by gender.

	OR (95% CI)	*P-*value	*P* for interaction
Cough			0.5023
Male	1.43 (1.28, 1.60)	< 0.001	
Female	1.36 (1.24, 1.50)	< 0.001	
Wheezing			0.2558
Male	1.69 (1.53, 1.87)	< 0.001	
Female	1.57 (1.44, 1.71)	< 0.001	
Dyspnea			< 0.001
Male	1.83 (1.69, 1.99)	< 0.001	
Female	1.44 (1.35, 1.54)	< 0.001	
COPD			< 0.001
Male	1.73 (1.50, 1.98)	< 0.001	
Female	1.26 (1.14, 1.40)	< 0.001	
Asthma			0.9774
Male	1.43 (1.27, 1.60)	< 0.001	
Female	1.43 (1.31, 1.56)	< 0.001	

Age, race, education level, marital status, PIR, and smoking were adjusted. Abbreviation: COPD, chronic obstructive pulmonary disease; OR, odds ratio; CI, confidence interval.

**Table 5:** Association between weight-adjusted waist index and respiratory symptoms, COPD, and asthma by race.

	OR (95% CI)	*P-*value	*P* for interaction
Cough			0.0748
Mexican American	1.19 (0.92, 1.52)	0.1829	
Other race	1.48 (1.15, 1.92)	< 0.01	
Non-Hispanic White	1.33 (1.20, 1.46)	< 0.001	
Non-Hispanic Black	1.66 (1.40, 1.98)	< 0.001	
Wheezing			< 0.05
Mexican American	1.40 (1.13, 1.73)	< 0.01	
Other race	2.13 (1.70, 2.66)	< 0.001	
Non-Hispanic White	1.59 (1.45, 1.75)	< 0.001	
Non-Hispanic Black	1.60 (1.40, 1.82)	< 0.001	
Dyspnea			< 0.05
Mexican American	1.52 (1.33, 1.74)	< 0.001	
Other race	1.95 (1.68, 2.26)	< 0.001	
Non-Hispanic White	1.59 (1.48, 1.70)	< 0.001	
Non-Hispanic Black	1.46 (1.32, 1.61)	< 0.001	
COPD			0.2795
Mexican American	1.21 (0.89, 1.66)	0.2291	
Other race	1.81 (1.35, 2.42)	< 0.001	
Non-Hispanic White	1.38 (1.24, 1.54)	< 0.001	
Non-Hispanic Black	1.39 (1.16, 1.66)	< 0.001	
Asthma			0.2695
Mexican American	1.15 (0.90, 1.47)	0.2689	
Other race	1.57 (1.26, 1.95)	< 0.001	
Non-Hispanic White	1.45 (1.31, 1.60)	< 0.001	
Non-Hispanic Black	1.47 (1.28, 1.70)	< 0.001	

Age, gender, education level, marital status, PIR, and smoking were adjusted. Abbreviation: COPD, chronic obstructive pulmonary disease; OR, odds ratio; CI, confidence interval.

### Smooth curve fitting and threshold effect analysis

**Fig 2:**
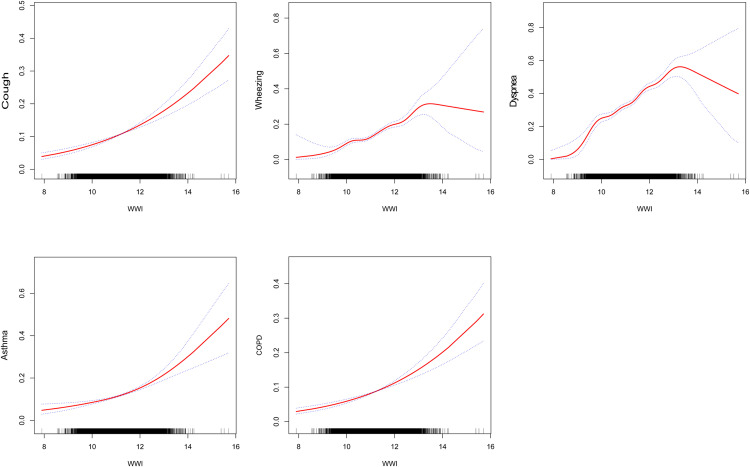
Relationship between WWI and incidence of respiratory symptoms, asthma, and COPD after adjusting for age, gender, race, education level, marital status, PIR, and smoking. Panel (A) shows the association between WWI and the incidence of cough, panel (B) shows the association between WWI and the incidence of wheezing, panel (C) shows the association between WWI and the incidence of dyspnea, panel (D) shows the association between WWI and the incidence of asthma, and panel (E) shows the association between BMI and the incidence of COPD.

Non-linear associations were identified between WWI and respiratory symptoms, including cough, wheezing, and dyspnea, as well as respiratory diseases such as COPD and asthma, employing smooth curve fitting and threshold effect analysis (Fig 2). J-shaped relationships were established between WWI and cough, COPD, and asthma, with OR of 1.39 (95% CI: 1.29–1.50, P < 0.001), 1.42 (95% CI: 1.30–1.54, P < 0.001), and 1.43 (95% CI: 1.33–1.54, P < 0.001), respectively (Table 6). A threshold effect was noted, indicating a breakpoint (K) of 9.99 for both wheezing and dyspnea (log-likelihood ratio < 0.05) (Table 6). In comparison to the right side of the breakpoint, where the odds ratios were 1.58 (95% CI: 1.47–1.70, P < 0.001) for wheezing and 1.55 (95% CI: 1.46–1.63, P < 0.001) for dyspnea, WWI on the left side of the breakpoint showed a significantly greater increase in the incidence of these symptoms with each unit increase in WWI, with odds ratios of 3.65 (95% CI: 1.55–8.56, P < 0.01) for wheezing and 3.07 (95% CI: 1.73–5.44, P < 0.001) for dyspnea.

**Table 6:** Threshold effect analyses between WWI and respiratory symptoms, COPD, and asthma.

	OR (95% CI)	*P*-value
Cough		
Standard logistic model	1.39 (1.29, 1.50)	< 0.001
Infection point (K)	9.99	
WWI < K	2.25 (0.88, 5.71)	0.0897
WWI > K	1.37 (1.26, 1.49)	< 0.001
Log-likelihood ratio		0.294
Wheezing		
Standard logistic model	1.62 (1.51, 1.73)	< 0.001
Infection point (K)	9.99	
WWI < K	3.65 (1.55, 8.56)	< 0.01
WWI > K	1.58 (1.47, 1.70)	< 0.001
Log-likelihood ratio		< 0.05
Dyspnea		
Standard logistic model	1.58 (1.50, 1.67)	< 0.001
Infection point (K)	9.99	
WWI < K	3.07 (1.73, 5.44)	< 0.001
WWI > K	1.55 (1.46, 1.63)	< 0.001
Log-likelihood ratio		< 0.05
COPD		
Standard logistic model	1.42 (1.30, 1.54)	< 0.001
Infection point (K)	12.3	
WWI < K	1.47 (1.33, 1.63)	< 0.001
WWI > K	1.10 (0.78, 1.57)	0.5875
Log-likelihood ratio	0.139	0.139
Asthma		
Standard logistic model	1.43 (1.33, 1.54)	< 0.001
Infection point (K)	12.18	
WWI < K	1.36 (1.25, 1.49)	< 0.001
WWI>K	1.82 (1.41, 2.36)	< 0.001
Log-likelihood ratio		0.056

Age, gender, race, education level, marital status, PIR, and smoking were adjusted. Abbreviation: WWI, weight-adjusted waist index; COPD, chronic obstructive pulmonary disease; OR, odds ratio; CI, confidence interval.

## Discussion

In this cross-sectional study involving 14,760 Americans aged 40 years or older, we found that higher levels of WWI were associated with an increased risk of respiratory symptoms and diseases. Smooth curve fitting revealed a nonlinear J-shaped association between WWI and the incidence of cough, COPD, and asthma. Furthermore, we identified a breakpoint (K) for wheezing and dyspnea at 9.99. For each unit increase in WWI, the incidence of wheezing and dyspnea was significantly higher at the left breakpoint (WWI = 9.99) compared to the right breakpoint.

In this cross-sectional study involving 44,480 U.S adults, utilizing NHANES data from 2001 to 2018, Yu et al. identified a positive correlation between WWI and the prevalence of asthma. Smooth curve fitting revealed a nonlinear relationship characterized by a threshold saturation effect, with an inflection point at 10.53 [29]. These findings generally align with our results; however, differences in inflection points may arise from variations in the populations studied. Sun et al. reported a U-shaped relationship between BMI, serving as an indicator of obesity, respiratory symptoms, and related diseases [20]. In contrast, our findings indicate a J-shaped relationship between WWI and respiratory symptoms, as well as related diseases, diverging from Sun et al.‘s observations. These discrepancies may be partly attributed to the ‘obesity paradox’ [30], a phenomenon that warrants consideration, as well as BMI’s limitations in distinguishing between fat mass and lean mass and its failure to account for localized fat distribution patterns [31]. Subgroup analyses by age, gender, and race were performed to evaluate the stability of the association between WWI and respiratory symptoms, along with associated respiratory diseases. The association between WWI and dyspnea and COPD exhibited significant variation by gender (P for interaction < 0.001), with males demonstrating a greater susceptibility to this correlation compared to females. Based on our findings, we hypothesize that variations in sex hormones contribute to disparities in fat metabolism and distribution patterns. Previous research has established a link between reduced testosterone levels and abdominal obesity in males, in contrast to the androgen excess observed in obese females [32]. Estrogen plays a role in the distribution of fat primarily in women’s thighs, hips, breasts, and other peripheral areas [33].

This study represents the first investigation into the relationship between WWI and respiratory symptoms, highlighting a positive association between WWI and the risk of developing COPD and asthma. WWI was utilized in this cross-sectional study to assess the level of ‘true obesity.’ The conclusions of our study are broadly applicable to the entire U.S population due to the use of nationally representative data with appropriate sample weights. Our analytical approach involved multifactorial logistic regression models that adjusted for several relevant covariates. Additionally, the substantial sample size enabled us to robustly validate our findings through subgroup analyses. However, it is crucial to acknowledge several limitations of our study. Firstly, the cross-sectional design precludes the establishment of causality between WWI and respiratory symptoms. Secondly, although we adjusted for multiple covariates, the NHANES database lacks comprehensive data on certain potential confounders, such as environmental exposures (e.g., air pollution or occupational hazards) and healthcare access (e.g., frequency of visits, regional disparities). These unmeasured factors may influence the observed associations. Thirdly, the dataset spans the years 2003–2012; while the underlying biological mechanisms and risk factors are unlikely to have changed significantly, potential shifts in environmental exposures and healthcare trends may affect the applicability of our findings to contemporary populations. Finally, regarding the generalizability of NHANES data, it is important to recognize both its strengths and limitations. Certain subpopulations, such as recent immigrants, individuals in rural or remote areas, and populations outside the U.S, may be underrepresented or inadequately captured. Additionally, cultural, environmental, and healthcare differences in non-U.S populations may limit the international applicability of these findings. While NHANES provides robust data for population-level analyses, future research in diverse populations and settings is needed to ensure broader generalizability.

## Conclusions

This study demonstrated a significant positive association between higher WWI and an increased risk of respiratory symptoms (cough, wheezing, and dyspnea) as well as respiratory diseases (COPD and asthma). Subgroup analyses revealed that these associations were influenced by age, gender, and race, while smoothed curve fitting suggested a J-shaped relationship for certain outcomes. Further research is needed to confirm these findings and explore the underlying mechanisms.

## Supporting information

S1 File The original data set for this study. (XLS)
